# Can Artificial Intelligence Replace Humans for Detecting Lung Tumors on Radiographs? An Examination of Resected Malignant Lung Tumors

**DOI:** 10.3390/jpm14020164

**Published:** 2024-01-31

**Authors:** Rurika Hamanaka, Makoto Oda

**Affiliations:** Department of Thoracic Surgery, Shin-Yurigaoka General Hospital, 255 Furusawa Asao-ku, Kawasaki 215-0026, Japan; rurika.hamanaka@gmail.com

**Keywords:** artificial intelligence (AI), chest radiograph, histopathological diagnosis, malignant pulmonary nodule, standardized uptake value

## Abstract

Objective: Although lung cancer screening trials have showed the efficacy of computed tomography to decrease mortality compared with chest radiography, the two are widely taken as different kinds of clinical practices. Artificial intelligence can improve outcomes by detecting lung tumors in chest radiographs. Currently, artificial intelligence is used as an aid for physicians to interpret radiograms, but with the future evolution of artificial intelligence, it may become a modality that replaces physicians. Therefore, in this study, we investigated the current situation of lung cancer diagnosis by artificial intelligence. Methods: In total, we recruited 174 consecutive patients with malignant pulmonary tumors who underwent surgery after chest radiography that was checked by artificial intelligence before surgery. Artificial intelligence diagnoses were performed using the medical image analysis software EIRL X-ray Lung Nodule version 1.12, (LPIXEL Inc., Tokyo, Japan). Results: The artificial intelligence determined pulmonary tumors in 90 cases (51.7% for all patients and 57.7% excluding 18 patients with adenocarcinoma in situ). There was no significant difference in the detection rate by the artificial intelligence among histological types. All eighteen cases of adenocarcinoma in situ were not detected by either the artificial intelligence or the physicians. In a univariate analysis, the artificial intelligence could detect cases with larger histopathological tumor size (*p* < 0.0001), larger histopathological invasion size (*p* < 0.0001), and higher maximum standardized uptake values of positron emission tomography-computed tomography (*p* < 0.0001). In a multivariate analysis, detection by AI was significantly higher in cases with a large histopathological invasive size (*p* = 0.006). In 156 cases excluding adenocarcinoma in situ, we examined the rate of artificial intelligence detection based on the tumor site. Tumors in the lower lung field area were less frequently detected (*p* = 0.019) and tumors in the middle lung field area were more frequently detected (*p* = 0.014) compared with tumors in the upper lung field area. Conclusions: Our study showed that using artificial intelligence, the diagnosis of tumor-associated findings and the diagnosis of areas that overlap with anatomical structures is not satisfactory. While the current standing of artificial intelligence diagnostics is to assist physicians in making diagnoses, there is the possibility that artificial intelligence can substitute for humans in the future. However, artificial intelligence should be used in the future as an enhancement, to aid physicians in the role of a radiologist in the workflow.

## 1. Introduction

Although lung cancer screening trials have showed the efficacy of computed tomography (CT) to decrease mortality compared with chest radiograph [1,2,3], chest radiographs are still widely taken in different kinds of clinical settings, such as outpatient clinics, for the investigation and follow-up of cardiopulmonary diseases. Furthermore, chest X-ray examinations are specified as an examination item in the general examinations based on laws and regulations in Japan, and many citizens undergo regular examinations in which pulmonary nodules are unexpectedly detected. Chest radiographs are not always checked by radiologists, and pulmonary nodules can also be missed even after checking by a radiologist. Lorentz et al. reported that the miss rate of 19% in their study was actually low compared with the rate in the literature [4]. Some previous studies reported that about 20% of lung cancers visible on chest radiographs were actually missed at the time of initial reading [4,5].

Artificial intelligence (AI) interpretation of chest radiographs is rapidly advancing and has been demonstrated to improve the performance of physicians at detecting lung cancer [5,6,7,8]. AI-based computer-assisted detection (CAD) of pulmonary nodules in chest radiographs has higher sensitivity especially, owing to the development of recent convolutional neural networks (CNN) [5,7,8,9,10] and deep learning (DL) algorithms. Shim et al. reported that the average sensitivity of radiologists improved from 65.1% to 70.3% and the number of false-positive findings per radiograph declined from 0.2 to 0.18 when the radiologists re-reviewed radiographs using the deep CNN software Samsung Auto Lung Nodule Detection (ALHD, version 1.00; Samsung Electronics, Suwon, South Korea) [7].

Currently, the interpretation of images by AI is still positioned as an aid to the interpretation of images by physicians [9,11,12], but if AI technology evolves further in the future, it may be possible that it will fully replace diagnoses by physicians. If image analysis by AI becomes the main diagnostic method, it will be advantageous for reducing human error, shortening examination times, reducing labor costs, and reducing litigation due to oversight.

Although AI will actually help doctors in cases where the radiologist cannot work, diagnoses based on chest radiography have been made by the attending physician at many hospitals in Japan, with additional readings by diagnostic radiologists being performed only when the attending physician made a request. Since November 2021, without additional requests from attending physicians, AI diagnostics has been performed for all cases undergoing frontal chest radiography at our hospital. Therefore, patients who undergo pulmonary resection for pulmonary malignant tumors at our hospital are evaluated using the current state of AI diagnostics for chest radiography before surgery. There are few reports that investigate in the clinical setting [13]. Our purpose in this study was to examine what types of tumors are likely to be overlooked by AI based on the histopathological characteristics of resected specimens and the maximum standardized uptake value (SUVmax) of positron emission tomography-computed tomography (PET-CT) and to ascertain the points to which physicians should pay attention to detect nodules on chest radiographs.

## 2. Patients and Methods

### 2.1. Ethics Statement

This study was conducted in accordance with the Declaration of Helsinki and its later amendments. This study was approved by the Ethics Review Committee of the Shin-Yurigaoka General Hospital, Kawasaki, Kanagawa, Japan (referral number: 20230424: approval date, 24 April 2023). The need for informed consent was waived because of the retrospective nature of this study.

### 2.2. Patients and Methods

In total, 174 consecutive patients who underwent pulmonary resection after AI diagnostics of at least one chest radiograph before surgery and were diagnosed with malignant lung tumors by postoperative histopathological diagnosis from November 2021 to July 2023 were included in this retrospective study. There were 96 male patients and 78 female patients. Age was 32–88 (median 73) years. Lung tumors were on the right side in 107 cases and on the left side in 67 cases. The localization of lung tumors on chest radiographs was in the lung apices field in 15 cases, the upper lung field in 63 cases, the middle lung field in 33 cases, the lower lung field in 53 cases, and the hilar area in 10 cases. Surgical procedures included 103 cases of robot-assisted thoracoscopic lobectomy, 35 cases of robot-assisted thoracoscopic segmental pulmonary resection, and 36 cases of thoracoscopic partial lung resection. The histopathological diagnoses were 130 cases of adenocarcinoma, 16 cases of metastatic lung tumor, 12 cases of squamous cell carcinoma, four cases of adenosquamous carcinoma, two cases of adenoid cystic carcinoma, two cases of neuroendocrine carcinoma, two cases of combined large cell neuroendocrine carcinoma, and two cases of combined small cell and squamous cell carcinoma.

### 2.3. AI-Based CAD Software

AI diagnoses of the nodules in chest radiographs in this study were made using the AI-based CAD software EIRL X-ray Lung Nodule version 1.12, (LPIXEL Inc.), which is commercially available in Japan as of August 2020 as a screening device to find primary lung cancer [9,14]. Our hospital introduced this software in November 2021 to detect lung nodules in all chest radiographs taken in the hospital. In principle, the EIRL X-ray Lung Nodule software version 1.12 aims to detect pulmonary nodules by determining areas that satisfy the following conditions: (1) 0.5 cm to 3 cm in size; (2) solid, not diffuse, shading; (3) close to circular in shape; (4) located in the thoracic cavity and not overlapping with other organs [9,14].

### 2.4. Statistical Analysis

SPSS software”Dr SPSS II for Windows based on SPSS version 11.0J for Windows, Tokyo, Japan) was used for statistical analyses, and the *t*-test was used to compare differences in mean values.

## 3. Results

AI diagnostics was performed once for 57 cases, twice for 13 cases, and more than three times for 104 cases. There were 90 cases in which an abnormality was pointed out at least once by the AI. All the 18 cases of adenocarcinoma in situ (AIS) could not be determined by AI (*p* < 0.0001).

We examined the sensitivity of the AI diagnostics regarding total size and invasive size (Table 1). The tumor sizes ranged from 0.4 cm to 6.1 cm (median: 2.1 cm), and the invasive sizes ranged from 0.0 cm to 6.1 cm (median: 1.7 cm). When comparing the tumor total size, the cases that could not be determined by the AI measured 1.8 cm on average, and the cases that could be determined by the AI measured 2.9 cm on average (*p* < 0.0001). When comparing the tumor invasive size, the cases that could not be determined by AI measured 1.2 cm on average, and the cases that could be determined by AI measured 2.5 cm on average (*p* < 0.0001).

We also examined the sensitivity of the AI diagnostics by nodule site in 156 cases, excluding cases of AIS. The sensitivity was 60% in the lung apices field, 63.6% in the upper lung field, 78.6% in the middle lung field, 43.8% in the lower lung field, and 30% in the hilar area. The detection rate for tumors in the lower lung field was significantly lower (*p* = 0.019) and the detection rate for tumors in the middle lung field was significantly higher (*p* = 0.014) than the detection rate for tumors in the upper lung field.

**Figure 1:**
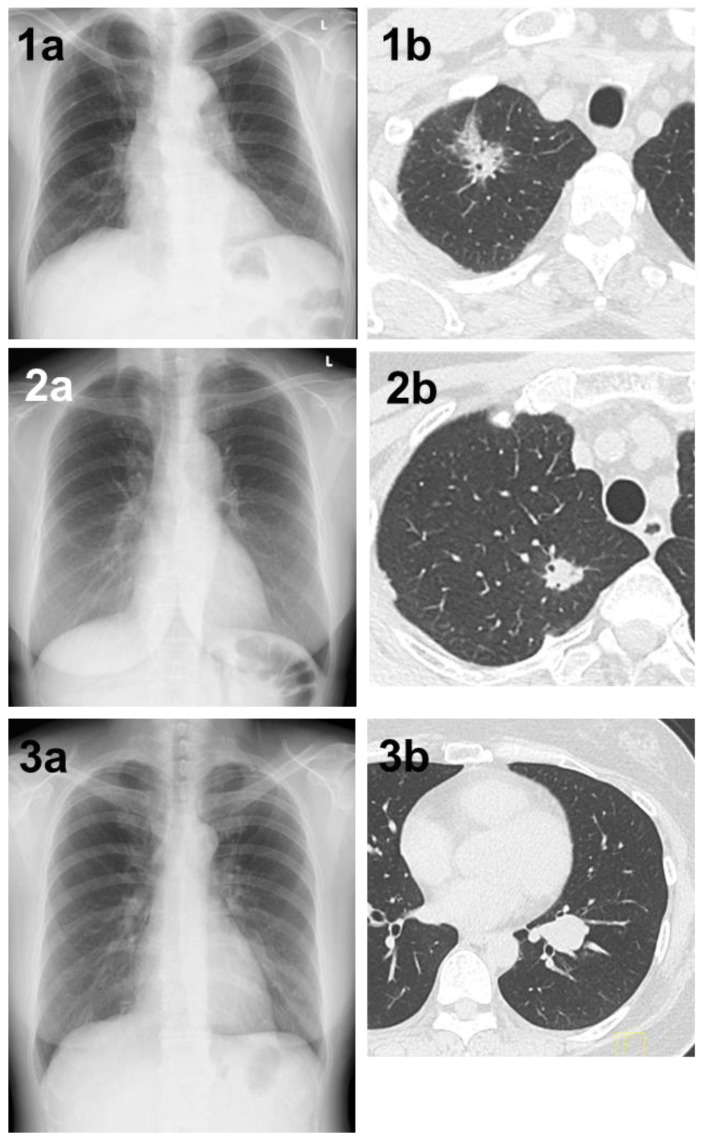
(**1a**) A shadow overlapping the clavicle in the right upper lung field that could not be determined by AI. (**1b**) Chest CT showing a mass with a total size of 4.3 cm and a solid size of 2.1 cm in the right S^1^ region. The histopathological diagnosis was adenocarcinoma (lepidic adenocarcinoma), total tumor and invasive size of 1.7 cm, pT1bN1M0 (stage IIB). (**2a**) A shadow overlapping the clavicle in the right upper lung field that could not be pointed out by AI diagnosis. (**2b**) Chest CT showing a nodule with a total and solid size of 1.6 cm in the right S^1^ region. The histopathological diagnosis was adenocarcinoma (lepidic adenocarcinoma), tumor total and invasive size 1.7 cm, pT1bN1M0 (stage IIB). (**3a**) A shadow that could not be determined by AI despite being a large tumor of 4 cm in diameter. A shadow running parallel to the blood vessels, partially overlapping the heart shadow in the left lower lung field. (**3b**) Chest CT showing a mass with a total and solid size of 4.3 cm in the left S^8–9^ region. The histopathological diagnosis was adenoid cystic carcinoma, total tumor and invasive size of 4.0 cm, pT2aN0M0 (stage IB).

**Figure 2:**
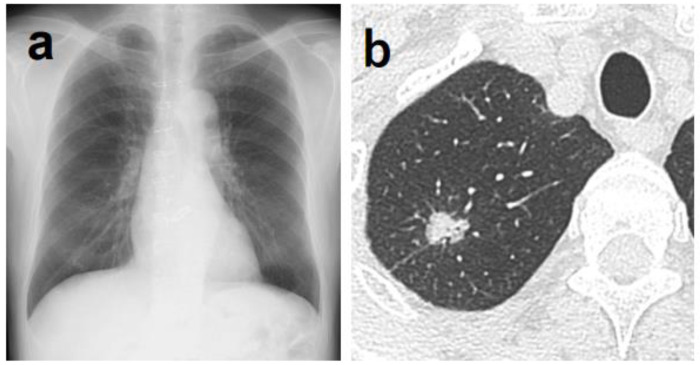
(**a**) Although it was difficult for physicians to determine the abnormal shadow, AI was able to highlight a shadow overlapping the clavicle in the right upper lung field. (**b**) Chest CT showing a nodule with a total and solid size of 1.6 cm in the right S^1^ region. The histopathological diagnosis was adenocarcinoma (papillary adenocarcinoma), total tumor and invasive size of 1.8 cm, pT1bN0M0 (stage IA2).

**Figure 3:**
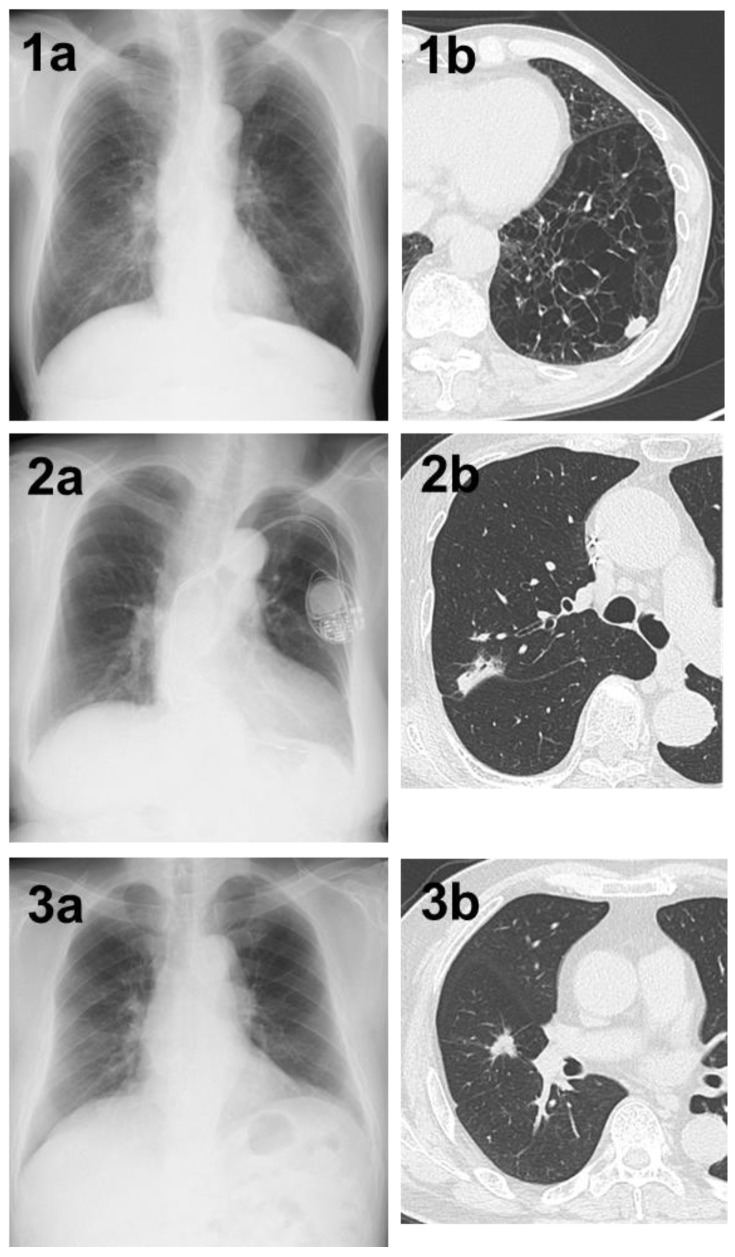
(**1a**) A shadow in the left lower lung field that was not determined by AI. (**1b**) Chest CT showing a nodule with a total and solid size of 1.5 cm in the left S⁹ region on the basis of strong emphysematous changes in the lung. The histopathological diagnosis was squamous cell carcinoma, total tumor and invasive size of 1.5 cm, pT1bN0M0 (stage IA2). (**2a**) A shadow in the right upper lung field that could not be determined by AI. (**2b**) Chest CT showing a nodule with a pleural indentation, total size of 2.8 cm, solid size of 2.3 cm, in the right S^2^ region. The histopathological diagnosis was adenocarcinoma (papillary adenocarcinoma), total tumor and invasive size of 3.4 cm, pT2aN0M0 (stage IB). (**3a**) A shadow in the right middle lung field that could not be determined by AI. (**3b**) Chest CT showing a nodule with a total and solid size of 1.5 cm in the right S⁴ region. The histopathological diagnosis was adenocarcinoma (acinar adenocarcinoma), total tumor size of 1.5 cm, invasive size of 1.4 cm, pT1aN0M0 (stage IA2).

The main reasons why lung tumors could not be detected by the AI in 84 cases are listed in Table 2. Thirty-five cases had lesions overlapping with anatomical structures, such as the mediastinum, heart, and clavicle, or lesions below the diaphragm. Eight of these 35 cases were detectable by physicians (Figure 1). Conversely, there was one case in which it was difficult for physicians to detect the shadow overlapping the anatomical structures, but it was detected by AI (Figure 2). Eighteen cases were AIS, thirteen were lepidic adenocarcinoma (invasive size: 0.05–2.0 cm), and six were small nodules less than 0.7 cm in diameter. The other six cases were difficult to visualize on chest radiographs. Of these six cases, three cases had ground-glass components as the main component on CT, and one case had nodule and pulmonary vessels visible in succession. Many of these lesions were difficult to visualize due to the imaging conditions. Six patients did not fit these conditions; their lesions could be determined by physician interpretation, and their non-detection was judged to be an oversight by the AI (Figure 3).

The sensitivity of the AI diagnosis was evaluated using the SUVmax of preoperative PET-CT (Table 3). We evaluated 166 patients, excluding eight who had not undergone PET-CT. The mean SUVmax value was 4.39 in cases where AI diagnosis could not detect the lesion and 8.82 in cases where AI diagnosis was possible (*p* < 0.0001).

A multivariate analysis of sex, age, tumor size, invasive size, and SUVmax of PET-CT revealed a significant difference only in invasive size of the tumor (*p* = 0.006) (Table 4).

## 4. Discussion

We performed a retrospective study to investigate the current clinical situation of lung cancer diagnosis by AI in our surgically treated patients. The fact that all the patients in this study were surgically treated offers the advantage that the histopathology and tumor invasion diameters were accurately known. The benefits of AI diagnostics in this study were as follows. The AI could significantly detect cases having large total histopathological tumor size, cases having large histopathological invasive size, and cases having high SUVmax of PET-CT. More-malignant tumors are often shadows that should not be overlooked on chest radiographs, and the fact that tumors with increased malignancy had a higher detection rate by the AI is a positive point for the clinical use of AI diagnostics.

Against these advantages, tumors in the lower lung fields were less frequently detected compared with tumors in the other lung fields, and physicians must pay careful attention to this area. Based on these results, AI diagnoses are currently considered to be appropriately positioned as a physician’s interpretation aid and as a second reader [6,9,11,12].

Commercially available AI-based computer-assisted detection software, such as Insight CXR, version 1.2.0.0 (Lunit, Seoul, Republic of Korea), can be used. In this study, the AI-based CAD software EIRL X-ray Lung Nodule version 1.12 (LPIXEL Inc.) was used as diagnostic tool for pulmonary nodules [9,14]. Ueda et al. reported that using this model with this software could support both general physicians and radiologists in the detection of lung nodules [9]. This CAD model increased physicians’ sensitivity with statistical significance without increasing the number of false positives [9].

In this report, the detection rate of pulmonary tumors excluding AIS by the AI was 57.7% (90/156 cases). It has been stated that smaller tumors are more difficult for physicians to detect via chest radiograph [4], and the same was true for the AI. Additionally, pure ground-glass nodules are difficult to detect on chest radiographs [15], but there were many cases in which AIS and lepidic adenocarcinoma were difficult to detect for both the AI and the physicians. It has also been reported that nodules smaller than 1 cm are below the resolution of chest radiographs, except for calcified nodules [15]. In the AI model we used, Shimizu et al. reported that the sensitivity was 0 for tumor diameters of 10 mm or less, 0.38 for 11–15 mm, 0.52 for 16–20 mm, and >83% for 21 mm or more [14]. Such nodules with small total or invasive size seem to be the limit for chest radiography.

Three other AI problems, as well as countermeasures and future issues, are discussed below. The first problem with the AI diagnostics was that it often missed shadows overlapping anatomical structures. Shadows that overlap with anatomical structures are often overlooked by the physician [4,8,15], and the same is true for AI. Additionally, it has been reported that upper lobe tumors are often overlooked by physicians [15], and there was a report that stated that this is a worldwide phenomenon [4]. In our study, the diagnostic rate of the AI was 43.8% in the lower lung field. It has been suggested that the lower lung field is affected by the fact that tumors in this area overlap with anatomical structures such as the liver, heart, and diaphragm. Using a DL AI model, Shimazaki et al. reported that sensitivity was lower in lung cancers that overlapped with blind spots, such as the pulmonary apices, pulmonary hila, chest wall, heart, and subdiaphragmatic space (0.50–0.64), compared with those in non-overlapped locations (0.87) [14]. Because the lung fields that are often overlooked by AI and physicians are different, it remains important to perform image interpretation by both AI and physicians as a measure to reduce oversights. Additionally, we believe that detection rates can be improved via devising ways to reveal hidden shadows in anatomical structures by changing the contrast of chest radiographs on the reading screen.

The second problem with the AI diagnostics was that it detected regions that satisfied the conditions listed above with the main purpose of detecting pulmonary tumors; thus, shadows inconsistent with the conditions may not be pointed out, even large shadows. The AI failed to identify a 4.0 cm adenoid cystic carcinoma in the bronchial lumen. Factors that could not be determined included the facts that the heart shadow and the nodule partially overlapped, that the tumor that developed in the lumen of the bronchi ran parallel to the blood vessel shadow, that the shape of the nodule and accompanying atelectasis was not circular, and that the lesion exceeded 3 cm. Because the AI determined that these lesions did not match the specified conditions, they were not highlighted. It is possible that three of the cases that were overlooked by the AI could not be detected because the tumors appeared flat on chest radiographs Malignant lung tumors do not necessarily appear as circular nodules on chest radiographs and may be suspected from the shape of atelectasis or infiltrative shadows around the tumor. The difficulty of diagnosing tumor-associated findings using AI is an issue for the future development of diagnostic AI algorithms. When there is a lack of radiologists in any area, in the clinical set up, general physicians should pay attention in their evaluation of chest radiographs using AI-based CAD assistance.

The third problem with the AI diagnostics was that in cases where AI diagnoses of chest radiographs were performed multiple times, the results varied each time. In this report, there were ten cases in which chest radiographs having tumors that could be detected by the AI were mixed with chest radiographs having tumors that could not be detected. Five cases included overlap with anatomical structures such as the clavicle and mediastinum. Regarding interpretations by physicians, it is common to make a comparative diagnosis if past chest radiographs are available, but the AI used in this study could not make a comparative diagnosis with past chest radiographs. AI with the added function of diagnosing with the help of a comparison with past images is also being developed and is expected to increase the accuracy of AI diagnoses.

If AI can detect all lesions that physicians can detect, AI diagnostics could replace humans. However, there are currently the problems mentioned above, and interpretation by physicians is still necessary [6,9,11,12]. As a solution, we believe that AI diagnostic technology can be improved by programming the lung nodule detection conditions in more detail. For example, lung cancer is often accompanied by typical changes in its surroundings (pleura indentation, vascular convergence, atelectasis on the peripheral side of the tumor), and we believe it would be useful to have AI learn these changes. Additionally, the imaging conditions of the chest radiography greatly affect the depiction of pulmonary nodules, so if it becomes possible to make a diagnosis via AIwhile performing appropriate gradation processing, we believe that this will lead to further improvements in AI diagnostics.

In a systematic review of the application of AI in diagnosing COVID-19 disease symptoms from chest X-rays, Kufel et al. concluded that (1) AI computational models used to assess chest X-rays in the process of diagnosing COVID-19 should achieve sufficiently high sensitivity and specificity, (2) their results and performance should be repeatable to make them dependable for clinicians, (3) these additional diagnostic tools should be more affordable and faster than the currently available procedures, (4) the performance and calculations of AI-based systems should take clinical data into account [16]. Although DL AI technology has been widely used recently to assess chest X-rays to detect pulmonary nodules, we should consider the same points.

Our study has several limitations. First, this study was a retrospective single-center study. Second, chest radiographs were usually checked by general thoracic surgeons and not routinely checked by radiologists in this study. Furthermore, we could not evaluate the difference in nodule detection rates among radiologists, a second intervention of radiologists after AI diagnosis, and general thoracic surgeons.

## 5. Conclusions

Our study showed that the diagnosis of tumor-associated findings and the diagnosis of areas that overlap with anatomical structures using AI is not satisfactory. In the future, AI is expected to be able to independently make diagnoses, offering benefits both from a medical standpoint by reducing labor costs and treatment times and from a legal standpoint by reducing lawsuits due to oversight. While the current standing of AI diagnostics is to assist physicians in making diagnoses, there is the possibility that AI can substitute for humans in the future. However, AI should be used in the future as an enhancement, to aid physicians in the role of a radiologist in the workflow.

## Figures and Tables

**Table 1:** Sensitivity of AI diagnosis by tumor total size and invasive size.

	Total Size			Invasive Size		
Size (cm)	Not Pointed Out by AI	Pointed Out by AI	Sensitivity (%)	Not Pointed Out by AI	Pointed Out by AI	Sensitivity (%)
−0.5	2	1	33.3	30	5	14.3
0.6–1.0	13	2	13.3	8	2	20.0
1.1–1.5	25	8	24.2	24	10	29.4
1.6–2.0	17	16	48.5	6	20	76.9
2.1–3.0	15	17	53.1	10	21	67.7
3.1–4.0	10	30	75.0	4	22	84.6
4.1–6.1	2	16	88.9	2	10	83.3

**Table 2:** Main reasons for 84 cases in which abnormal shadows could not be detected by AI diagnosis.

Cause	Number of Cases
Overlapping anatomical structures	35
AIS	18
Lepidic adenocarcinoma	13
Oversight (can be detected in interpretation by a doctor)	6
Small nodules (≤0.7 cm)	6
Others	6

**Table 3:** Sensitivity of AI diagnosis by SUVmax of PET-CT.

SUVmax	Not Pointed Out by AI	Pointed Out by AI	Sensitivity (%)
0.00	30	4	11.8
0.01–3.00	14	12	46.2
3.01–5.00	8	18	69.2
5.01–10.00	16	24	60.0
10.01–15.00	7	17	70.8
15.01–	3	13	81.8

**Table 4:** Examination by multivariate analysis of significantly associated factors.

Variables	*p*	HR	95%CI
Sex	0.437	0.745	0.354–1.565
Age	0.434	0.988	0.957–1.019
Tumor size	0.310	1.026	0.976–1.080
Invasive size	0.006	1.081	1.022–1.142
SUVmax	0.514	1.023	0.956–1.095

## Data Availability

The data underlying this article will be shared upon reasonable request to the corresponding author.

## Abbreviations

AI—artificial intelligence; AIS—adenocarcinoma in situ; CT—computed tomography; CAD—computer-assisted detection; CNN—convolutional neural networks; DL—deep learning; PET-CT—positron emission tomography-computed tomography; SUVmax—maximum standardized uptake value.
